# The Management of Psychiatric Emergencies in Situations of Public Calamity

**DOI:** 10.3389/fpsyt.2021.556792

**Published:** 2021-01-28

**Authors:** Leonardo Baldaçara, Antônio Geraldo da Silva, Lucas Alves Pereira, Leandro Malloy-Diniz, Teng Chei Tung

**Affiliations:** ^1^Associação Brasileira de Psiquiatria, Rio de Janeiro, Brazil; ^2^Medicine, Universidade Federal do Tocantins, Palmas, Brazil; ^3^Faculdade de Medicina, Universidade do Porto, Porto, Portugal; ^4^Departamento de Psiquiatria, Escola Bahiana de Medicina e Saúde Pública, Salvador, Brazil; ^5^Mental Health Department, Universidade Federal de Minas Gerais, Belo Horizonte, Brazil; ^6^Psychology Department, Universidade FUMEC, Belo Horizonte, Brazil; ^7^Universidade de São Paulo, São Paulo, Brazil

**Keywords:** humans, pandemics, psychiatric emergencies, mental health, outbreaks

## Abstract

The prevalence of mental health problems in the general population during a public calamity is high. In calamities, the number of patients who present with mental disorder outbreaks or crises may increase, but the necessary support systems to help them may be impaired if they have not been planned for. Although there are several models for addressing psychiatric emergencies, the general rules are the same, especially when it comes to making these services easily available to the affected population. In this article, we seek to review and present recommendations for the management of psychiatric emergencies in situations of public calamity, including disasters, physical and medical catastrophes, epidemics, and pandemics.

## Introduction

To understand the effects of medical emergencies in public health, we can use various human calamities on a large scale as an example (1). These include disasters, physical and medical catastrophes, epidemics, and pandemics, such as the current outbreak of COVID-19 (2–4). Analysis of the relationship between natural catastrophes and human behavior is crucial in understanding both how to deal with its impact and consequences and how to manage its effects on mental health (3, 5).

In turn, the prevalence of mental disorders in disaster situations is two to three times higher than that in normal situations and varies from 8.6 to 57.3% in the affected population (6, 7). In addition, the affected community can exhibit many subsyndromal symptoms. Many reactions and acute disorders can be self-limiting, while others may require specialized assistance (7).

Nonetheless, one of the most critical issues concerning the effects of natural catastrophes is their impact on mental health (2, 3, 8), including the worsening of symptoms in patients and increases in psychiatric emergencies (1, 9, 10). Psychiatric disorders, such as posttraumatic stress disorder, anxiety, and depression, have frequently impacted both survivors directly affected by a calamity and health professionals who worked during the crisis (6, 9, 11, 12). In addition to the long-term consequences of calamities, the strategies used to address emergencies in health care during the acute crisis are crucial in minimizing their immediate long-term impact.

The problems discussed here can affect hospitals several times during a given period. A decrease in the number of employees and an increase in patients may temporarily compromise the hospital's service capacity, which makes it impossible to prevent bad outcomes that would have been avoidable previously (1). Hospitals must close their doors and divert patients as the first step. If hospitals have a general duty to be open to the health care needs of incoming patients, outbreaks and staff shortages can justify the diversion of ambulances to other nearby centers. Despite this delay, the goal is still to prevent bad outcomes using all available medical and nursing resources (1–3).

Medical emergencies are situations where the individual is at risk of imminent death; thus, they require immediate intervention. Changes in a patient's behavior that put them or others at risk and that require immediate therapeutic intervention (in a matter of minutes or a few hours) to prevent harm are called psychiatric emergencies. The most prevalent emergency situations are severe self-neglect, self-harm, suicidal behavior, depressive or manic episodes, aggressive psychomotor agitation, severely impaired judgment, intoxication, or withdrawal from psychoactive substances (13, 14).

In calamities, the number of patients in outbreak or crisis situations may increase, but the necessary support may be impaired if it has not been planned for (2, 3, 10). Although there are several models for addressing psychiatric emergencies, the general rules are the same, especially when it comes to making these services easily available to the population (10).

This review aims to propose strategies to address psychiatric emergencies in calamity situations and includes the discussion of attitudes toward public policies and codes of conduct for health professionals.

## Search Strategy and Selection Criteria

This is a review of the management of psychiatric emergencies in situations of public calamity. To identify relevant studies, we searched MEDLINE. The search terms were psychiatric emergencies and calamity or disasters or epidemic or pandemic. We applied no language or time restrictions. We supplemented the search results with important publications.

The main recommendations for agitation management are present in Table 1. In Tables 2–5 we present main recommendations for some medications that could be used in emergencies. In Table 6 we present key points about psychiatric emergencies in public calamities.

**Table 1:** Management of psychomotor agitationin public calamities.

Assess the situation. Protect the patient, people, and staff
Use verbal de-escalation and other communication techniques
Move the patient to a more peaceful environment
Use rapid oral tranquilization
Recommended antipsychotics: Asenapine, haloperidol, risperidone, olanzapine. Avoid use in patients with arrhythmia, seizures, and head trauma Recommended benzodiazepines^*^: Clonazepam, diazepam (especially for alcohol withdrawal), and lorazepam. Avoid use with SNC depression drugs (i.e., alcohol)
Use rapid intramuscular tranquilization
Recommended antipsychotics: Aripiprazole, haloperidol, olanzapine. Avoid use in patients with arrhythmia, seizures, and head trauma Recommended benzodiazepines: Lorazepam and midazolam. Avoid use with SNC depression drugs (i.e., alcohol)
Use physical restraint with rapid tranquilization

* *IV must be avoided. Exceptions are alcohol withdrawal, cocaine intoxication, and seizures where IV diazepam or lorazepam is indicated*.

**Table 2:** Main warnings for antipsychotics.

**Medication**	**Warning**
Typical—Low potency	Drug interactions: Antihypertensives, antiarrhythmic medications, sedatives, other antipsychotics, ciprofloxacin, fluvoxamine, lithium, clinafloxacin, idrocilamide, oltipraz, rofecoxibe, etintidine, zafirlukast, chloroquine/hydroxychloroquine, ritonavir, azithromycin Diseases: CNS depressor intoxication (alcohol, psychotropic medications), dementia, cardiovascular disease, head injury, anticholinergic effects, hematologic toxic, liver disease, parkinsonism, renal dysfunction, respiratory disorders, seizures, thromboembolic events, seizures
Atypical—High potency	Drug interactions: Antihypertensives, antiarrhythmic medications, fluoxetine, sedatives, other antipsychotics, venlafaxine, escitalopram, divalproex, lithium, ondansetron, erythromycin, levofloxacin, moxifloxacin, pentamidine, toremifene, vandetanibe, rifampicin, itraconazole, chloroquine/hydroxychloroquine, ritonavir, azithromycin Diseases: CNS depressor intoxication (alcohol, psychotropic medications), dementia, cardiovascular disease, head injury, anticholinergic effects, hematologic toxic, liver disease, parkinsonism, renal dysfunction, respiratory disorders, seizures, thromboembolic events, dehydration, hyperthyroidism, neutropenia, seizures
Atypical	Drug interactions: Antihypertensives, antiarrhythmic medications, sedatives, other antipsychotics, metoprolol, lamotrigine, SSRI, lithium, pregabalin, carbamazepine, clonazepam, cimetidine, ketoconazole, rifampicin, ciprofloxacin, chloroquine/hydroxychloroquine, ritonavir, azithromycin, iodinated contrasts Diseases: CNS depressor intoxication (alcohol, psychotropic medications), dementia, cardiovascular disease, head injury, anticholinergic effects, hematologic toxic, liver disease, parkinsonism, renal dysfunction, respiratory disorders, seizures, thromboembolic events, hypotension, anticholinergic effects, seizure, aspiration, lipid alterations, hematologic abnormalities (clozapine: agranulocytosis), diabetes

**Table 3:** Main warnings for mood stabilizers.

**Medication**	**Warning**
Lithium	Drug interactions: Acyclovir, angiotensin converting enzyme inhibitors, digoxin, diuretics, non-hormonal anti-inflammatory drugs, tricyclic antidepressants, beta blockers, chloroquine/hydroxychloroquine, ritonavir, lopinavir, streptomycin, phenytoin, levodopa, antipsychotics, tapazole, tenofovir, tetracycline, warfarin Diseases: Renal diseases, seizures, hypothyroidism, cardiac disease, diarrhea, sodium depletion, fever, neutropenia, dehydration
Valproate	Drug interactions: Antiretrovirals, antithyroid drugs, calcium channel blockers, carbamazepine, carbapenems, cimetidine, chloroquine/hydroxychloroquine, clozapine, chemotherapy, dapsone, digoxin, erythromycin, ethosuximide, phenytoin, phenobarbital, lamotrigine, ritonavir, salicylic acid, lopinavir, tricyclic antidepressants, warfarin Diseases: Liver disease, HIV/CMV, thrombocytopenia, renal disease, thyroid diseases
Lamotrigine	Drug interactions: Carbamazepine, citalopram, clozapine, desmopressin, phenytoin, phenobarbital, chloroquine/hydroxychloroquine, ritonavir, lopinavir, rifampicin, sulfamethoxazole-temoporfin Diseases: Rash, arrhythmias, blood dyscrasias, meningitis, renal disease, liver disease

**Table 4:** Main warnings for antidepressants.

**Medication**	**Warning**
Agomelatine	Drug interactions: Antidepressants, ciprofloxacin, betablockers, estrogens Diseases: Cutaneous reactions, hepatitis
Bupropion	Drug interactions: Alprazolam, aripiprazole, amphetamines, dextroamphetamine, citalopram, clonazepam, duloxetine, venlafaxine, lamotrigine, escitalopram, linezolid, lisinopril, paroxetine, fluoxetine, quetiapine, tramadol, trazodone, sertraline, ritonavir, efavirenz, betablockers, cyclophosphamide, tamoxifen, ticlopidine, clopidogrel, aminophylline theophylline Diseases: Heart diseases, seizures, glaucoma, hypertension, head trauma, delirium
Dual-action antidepressants	Drug interactions: Antidepressants, antihypertensives, cimetidine, clozapine, diuretics, lithium, metoclopramide, ondansetron, oxcarbazepine, phenytoin, tramadol, quinolones, tamoxifen, triptans, warfarin Diseases: Heart diseases, seizures, glaucoma, hypertension, head trauma, delirium
Mirtazapine	Drug interactions: Antidepressants, buspirone, sedatives, ketamine, ketoconazole, cimetidine, clonidine, erythromycin, phenytoin, hydroxyzine, levodopa, metoclopramide, olanzapine, propranolol, quetiapine, risperidone Diseases: Renal disease, liver disease, hypotension, neutropenia, hyponatremia, seizures, glaucoma
Monoamine oxidase inhibitors	Drug interactions: Adrenalin and sympathomimetic amines, anesthetics, antihistamine, antidepressants, caffeine, carbamazepine, cyproheptadine, clonidine, clozapine, decongestants, disulfiram, ginseng, insulin, levodopa, lithium, methyldopa, opioids, nitrates, oxcarbazepine, tyramine, triptans, tryptophan Diseases: Heart disease, cerebrovascular disease, use of sympathomimetics, thyrotoxicosis, pheochromocytoma, liver disease, renal disease
Selective serotonin reuptake inhibitors	Drug interactions: Amiodarone, antidepressants, betablockers, ciclosporin, cimetidine, clarithromycin, digoxin, fluconazole, glibenclamide, insulin, linezolid, aspirin, lorazepam, ibuprofen, lamotrigine, gabapentin, omeprazole, opioids, oral anticoagulants, ritonavir, triptans, tryptophan, warfarin, verapamil Diseases: Hyponatremia, seizures, QT prolongation, SIADH, liver disease, platelet function, renal disease, glaucoma, diabetes
Trazodone	Drug interactions: Amiodarone, antidepressants, antihypertensives, clonidine, digoxin, Ginkgo Biloba, linezolid, methyldopa, propranolol, ritonavir, selegilin, warfarin Diseases: Glaucoma, seizures, cardiovascular disease, hyponatremia, hypotension, liver disease, renal disease
Tricyclics	Drug interactions: Acetaminophen antihistamines, bupropion, duloxetine, cyclobenzaprine, gabapentin, opioids, lamotrigine, escitalopram, pregabalin, fluoxetine, levothyroxine, linezolid, sertraline, topiramate, trazodone, sulfamethoxazole trimethoprim, fluconazole Diseases: Anticholinergic effects, cardiovascular disease, seizures, glaucoma, urinary retention, neutropenia, bone marrow suppression
Vortioxetine	Drug interactions: Abciximab, anticoagulants, antidepressants, betablockers, buspirone, carbamazepine, efavirenz, protease inhibitors, linezolid, lithium, lopinavir, opioids, ritonavir

**Table 5:** Main anxiolytics for acute anxiety symptoms^*^^,^^**^^,^^***^.

Alprazolam	±11.2	Bupropion, cyclobenzaprine, furosemide, gabapentin, lisinopril, losartan, omeprazole, trazodone
Bromazepam	±17	Antifungals, macrolide antibiotics, HIV protease inhibitors, calcium channel blocking agents
Clonazepam	±35	Aripiprazole, antifungals, bupropion, duloxetine, fluoxetine, phenytoin, ranitidine, phenothiazines, monoamine oxidase carbamazepine, sertraline, pregabalin, lamotrigine, phenobarbital
Diazepam	±48	Baclofen, duloxetine, furosemide, cimetidine, lisinopril, omeprazole, prednisone, omeprazole, sertraline
Lorazepam	±18	Aripiprazole, citalopram, duloxetine, escitalopram, pregabalin, bupropion, sertraline

* *Avoid use with Antihistamines, opioids, and other sedatives. Avoid use in acute alcohol intoxication, closed-angle glaucoma, drug dependence, renal/liver disease, respiratory depression seizures, prolonged hypotension*.

** *Drug interactions and/or related problems: Cimetidine, oral estrogen-containing contraceptives, diltiazem, disulfiram, erythromycin, fluoxetine, fluvoxamine, grapefruit juice, itraconazole, ketoconazole, nefazodone, propoxyphene, ranitidine, and verapamil, which inhibit the oxidative metabolism of benzodiazepines, are less likely to affect lorazepam, which undergoes glucuronide conjugation*.

*** *Be careful in cases of depression, obesity, and in the presence of paradoxical reactions*.

**Table 6:** Key points about psychiatric emergencies in public calamities.

Psychiatric emergencies are complications of mental illnesses that may be pre-existing or arise after the event
For this reason, full support services must be available including outpatient care, day hospitals, emergency services, and general and specialized wards
It is necessary for the population to have quick access to screening and rapid referral mechanisms during and after a disaster
The assessment must be complete and consider the differential diagnosis or comorbidities with physical diseases
Main emergencies include psychomotor agitation, suicide behavior, substance abuse, mood disorders, psychosis, and anxiety disorders

## Assurances to the Affected Population

The population affected by the calamity should receive general health care and support for psychiatric and mental health services. According to Ho et al. (15), in the COVID-19 pandemic crisis, it is important to use certain strategies to deal with a high-risk population under stress (e.g., foreigners under quarantine). They also argue that mental health care should include screening scales to assess the impact of outbreaks on mental health in people with frequently described COVID-19 symptoms and people with a history of psychiatric symptoms (15). In addition to the assistance provided by the emergency services specialist (which includes places for observation), access to outpatient treatment must also be guaranteed to affected individuals, since this provides care for mild cases and prevents future episodes or crises (16). Places for hospitalization must be guaranteed for the treatment of acute cases (2, 3). Mobile prehospital emergency care must be made available, and their staff must be trained to deal with psychiatric emergencies (2, 17, 18). All these settings should be supported by psychiatrists (10, 19).

Other tools should be made available to the population, both for screening acute cases and for support and prevention (16, 17, 20), and should include the following:

- Clear mechanisms to triage and referral (17)
- Psychosocial evaluation and recognition of vulnerable populations (2, 17, 20)
- Effective communication to the affected population (3, 17, 20)
- Assessment of psychopathology and psychophysiology of fear and related behavioral responses, focusing on psychiatric emergency events (17, 20)
- Assessment of cultural context of mental health (17, 20)
- Psychological first aid (20)
- Caring for the healthcare and support workers' mental state (17, 20)
- Services for differentiating normal distress from pathological stress (20)
- Digital access to relevant mental health information, support, and intervention (3, 12)

Telepsychiatry can be used as a support tool to provide care in places that are difficult to access. However, it is necessary to consider its cost effectiveness, the availability of equipment on site, and any related legal aspects. One caveat is that telepsychiatry should only be used as an instrument to mitigate the lack of stable patient care and thus prevent crises, but it is not recommended for addressing ongoing psychiatric emergencies.

## Structure of Care

Psychiatric emergencies can occur anywhere and unexpectedly. Therefore, it is necessary to emphasize that the appropriate environment for dealing with crises will not always be available to health professionals (10, 19, 21, 22). The safety of the patient and those close to them should be the initial concern in the management of psychomotor agitation. Both the doctors and other team members should not put themselves in risky situations, such as attending the patient in a closed room with no accessible exit or attending them without personal safety equipment (13, 23, 24).

An adequate physical structure is essential for assisting patients in emergency situations. Psychiatric patients should be treated in a specific physical area reserved for such care, given the peculiarities of the clinical presentations of mental illness. The location must have adequate physical space for the nursing staff to provide specific care, with, for example, well-ventilated offices and bathrooms at the location (13, 21, 25, 26). There should be adequate lighting and guidance items, such as clocks and calendars, to help those who are confused (26). In the waiting room, the furniture must be organized, and the office must have easy access points for the patients and for healthcare professionals (21).

It is essential to ensure that patients are comfortable and to minimize external stimuli. Loud sounds, bright colors, and excessive heat or cold are inappropriate sensory stimuli and can aggravate some psychiatric symptoms. Care must be taken to ensure that patients are comfortable and that external stimuli are minimized. The psychiatric emergency department has adequate facilities for both entry and exit. Rooms must be quiet and individual, and waiting times must be minimized as much as possible (21, 24–28).

In the room used for attending psychiatric emergencies, there should be chairs and a table for the patient and family, a table for examination, and a sink for washing hands (13, 26, 29). The exit route from the room must be at the back of the professional who attends the patient and should be completely unobstructed so that it can be used in the case of a threat that cannot be managed (29, 30). It is important to note that doctors and other health professionals will care for patients who may be in crisis and who may behave in an unpredictable manner (29, 30). Objects that are potentially dangerous should always be removed (24, 28).

Psychiatric emergencies often have an organic etiology, and clinical complications inherent to the illness or treatment can occur. It is essential that emergency equipment, such as oxygen tanks, equipment for orotracheal intubation, secretion aspirators, vaporizers and nebulizers, carts, and trays with defibrillators, are easily accessible (26). Laboratory tests, such as capillary blood glucose tests, oximetry, and ECG (24), should be available on site. Materials for physical restraint, such as appropriate ranges, must also be available (13, 21, 26, 30).

The observation areas should be equipped with beds with raised heads and fixed bars where restraints may be attached if necessary. It is inadvisable that patients under observation remain on stretchers (26). The layout must be organized to facilitate observation with unobstructed lines of sight, and all blind spots must be eliminated (25). All services to handle behavioral changes must be provided in an area or room designed to reduce patient agitation (25).

To reduce the risk of transmission of infectious diseases, such as H1N1, COVID-19, or tuberculosis, health professionals who care for patients in psychiatric emergencies must be prepared for protective measures, including the provision of isolation areas, containment protocols, and the availability of personal protective equipment, whether they are in an emergency room or not. During an epidemic, the management of behaviorally aggressive or hostile patients will always be a difficult challenge, since it would not be clear if the agitated/aggressive patient is infected or not, so any psychiatric agitated patient should always be managed as a possible infected case.

Consideration should be given to opening observation areas and exclusive wards for mentally ill patients during an epidemic, to contain transmission or deal with emergency situations (such as agitation). The health care team should always be dressed and prepared for immediate intervention. In addition, many people with mental illness may not have the capability to understand preventive measures, such as frequent hand washing, wearing masks, and physical distancing.

The number of patients must not exceed the number of beds available, given that an excess number of patients can increase the tension between patients and staff (25). Whenever the management team engages with patients exhibiting agitated and violent behaviors, an effort must be made to manage the treatment in a less restrictive physical environment (25), such as in a small specialized observation unit with adequate space, equipment, security, and trained staff (13, 25, 30, 31). This is a small, specialized observation unit with suitable space, equipment, safety, and trained teams (25, 31). The Psychiatric Intensive-care Unit (PICU) has showed better results than even a psychiatric department specializing in the care of acute patients (32).

## Staff

The team should be trained, and there should be protocols for the therapeutic approach of the main psychiatric emergencies. These protocols provide each step of the approach to the patient and the role of each professional to that extent (13, 26).

Physicians working in intensive care settings must have the ability to perform multiple tasks simultaneously and tolerate rapid changes in patients' priorities (28). In this environment, it is important to tolerate and even enjoy dealing with agitated patients. This requires certain characteristics of temperament, and all doctors are encouraged to self-assess their own temperament for this kind of work (28). Agitated patients can be provocative and may challenge the clinician's authority, competence, or credentials. Some patients, to deflect their own sense of vulnerability, are very sensitive in detecting the clinician's vulnerabilities and thus focus on them (28).

It is necessary for everyone to assume a role in this type of service. It is necessary to wear appropriate clothing, such as a lab coat or non-provocative clothing in neutral colors and badges. Drop earrings, necklaces, or long hair are not recommended. The reason for such recommendations is to minimize or even avoid the possibility of being the victim of an attack by a more aggressive patient (13, 21, 33). Sudden movements and prolonged direct eye contact can be perceived as a threat and should be avoided. An adequate distance must be maintained from agitated patients to protect the team and the patient (21, 33).

In calamity situations involving infectious diseases, emergency professionals may suffer from interpersonal isolation and fear of transmitting the virus to their families. Medical teams have also stated that wearing protective clothing such as N95 masks can hinder communication between team members and with patients. During Korea's 2015 MERS-CoV outbreak, the effects of stigma and hardship had a direct impact on the mental health of health professionals who worked in public hospitals (34, 35). Psychological adaptation was described among health personnel who had access to well-equipped and structured environments (34).

Medical workers in Wuhan have been dealing with a high risk of infection, inadequate protection against contamination, overwork, frustration, discrimination, isolation, patients with negative emotions, a lack of contact with their families, and exhaustion in COVID-19 outbreak (2, 18, 34, 36). The current situation is causing mental health problems such as stress, anxiety, depressive symptoms, insomnia, denial, anger, and fear (34, 37). These mental health problems not only affect the attention, understanding, and decision-making capacity of medical workers, which could hinder the fight against COVID-19, but they could also have a lasting effect on their overall well-being of professionals (2, 18, 34, 36) and can result in an expected increase in cases of posttraumatic stress (PTSD).

Then, assistance to provide enough equipment, protocols, and psychological and psychiatric support is essential, since this will preserve the proper functioning of the team and, in turn, improve patient care.

## Assessment

When patients and family members go to the emergency department due to any psychiatric emergency, one must quickly and effectively attempt to analyze the situation to implement the best treatment as soon as possible. The protocol to care for patients in psychomotor agitation can be extrapolated to other emergencies, since the main goals are screening and severity assessment (13, 21, 24, 30, 38, 39), and includes the following:

a. Objective and subjective anamnesis.
b. Physical and neurological examination.
c. Psychiatric examination.
d. Differential diagnosis.
e. Quick tranquilization.
f. Referral and orientation.

In some cases, it may be difficult to perform all these steps as soon as patients are seen by health workers. Because time is important in an emergency, we suggest the following four basic questions (13, 29):

A. *What is happening?*

Investigate what behavioral changes are observed that are of concern. It is important to determine the acute changes in behavior that can put the patient or others at risk and eliminate any organic causes.

B. *For how long?*

Investigate whether the patient has experienced such changes for a long or short period and confirm whether there have been any serious and acute changes in behavior. Even in the case of patients with a long history of agitation, the situation can be urgent. Sudden changes in behavior can also be caused by organic factors.

C. *Why today?*

Investigate why they chose this moment to seek help. Some crucial triggering factors may be considered circumstantial or irrelevant or simply were not mentioned by the patient or relatives.

D. *What is the diagnostic hypothesis or temporary diagnosis?*

Start the differential diagnosis process to identify the best approach. In the emergency room, syndromic diagnoses, such as psychotic disorder or mood disorder, are preferably used since the need for quick decision making will not allow a detailed diagnosis.

## Approach to the Main Situation

### General Support for Stress

As mentioned earlier, it is necessary for the population to have quick access to screening and rapid referral mechanisms during and after a disaster. Subsequently, patients should receive care focused on possible mental disorders (pre-existing) and for situations that represent a greater vulnerability to stress. This is especially true for people who exhibit an abnormal response to the disaster or calamity (7) such as the following:

- Survivor's guilt,
- Becoming mentally ill,
- Stress related to caring for a person with physical or mental illness,
- Fear of losing control of overwhelming emotions,
- Substance use,
- Death wishes and suicidal ideas. Through emotional validation, a sense of justification can be provided for these overwhelming emotions.

Some interventions suggested for these abnormal responses to a calamity are as follows:

- *Psychological First Aid*: Survivors can exhibit many physical, emotional, and cognitive symptoms. The patient may not be able to think and act rationally during the disaster. Psychological first aid techniques can be performed by non-professionals who have been minimally trained within the community (7, 40).
- *Crisis-focused intervention/psychotherapy*: The focus of this intervention is to stabilize patients, interrupt distress escalation, mitigate acute signs and symptoms, restore functionality, and establish bonds and agreement on therapy goals. This type of intervention is useful for the treatment of entrapment sensations, which are frequently observed in psychiatric emergencies (41).
- *Debriefing:* This is defined as group discussions that occur within 48–72 h after an event and are often referred to as ‘psychological debriefings' (7). These sessions encourage participants to describe and share both factual and emotional aspects of their disaster experience (7, 11). The justification is that immediate processing gives an individual the ability to cognitively restructure the perceived disaster event so that it is remembered in a less traumatic way (7).
- *Cognitive Behavioral Intervention (CBT):* CBT has been found to be effective in reducing subsequent psychopathology after exposure to a disaster (7, 11). In emergencies, CBT should be used in brief sessions and includes a range of techniques, such as psychoeducation, breathing exercises, relaxation exercises, and cognitive restructuring. Techniques that address traumatic experiences should include imagery and/or *in vivo* exposure (42).
- *Community-Based Interventions:* These interventions include the structuring of daily activities to avoid displacement; promotion of family, cultural, and religious rituals; group discussions; validation of the survivor's emotions and the survivor's guilt; provision of factual information; education of parents and teachers; involvement of children in various informal methods of education with innovative ideas, such as drawing, singing, imitating, and so on, using available community resources; and involvement of adult survivors in activities at a disaster camp, such as cooking, cleaning, and helping with relief work. Schools in the area affected by the disaster should be re-opened as early as possible, so that the normalization and structuring of daily activities can occur in children, even if they just receive some informal education, learn simple sleep hygiene techniques, and are educated about the harmful effects of substance use. Community-based group interventions can be planned as well and include art therapy (painting/drawing), group discussions, dramas, narratives, planning of daily routines, and participation in activities, prayers, yoga, relaxation, and sports/games. While managing social worker stress is essential, it is also essential to involve willing survivors in spiritual activities and to involve them in rebuilding their community (7).
- *Psychopharmacology:* Generally, the use of psychotropic drugs is discouraged in disasters because of popular notions such as “disaster reactions are usually normal people in abnormal situations” and “most symptoms are self-limiting.” Prophylactic use of psychotropic drugs in survivors is often discouraged. There are no well-controlled studies to show that prophylactic drug use decreases psychiatric morbidity (7). The exception is when there is a diagnosis of mental disorder that may have arisen or worsened during the calamity, but in these cases, the treatment is aimed at the specific diagnosis.

### Delirium

Delirium is a syndrome where there is mental confusion characterized by impaired consciousness, cognitive function, and attention, with an abrupt onset and a fluctuating course. It is associated with a rapid reduction in brain function and is caused by physical illnesses, usually of systematic involvement. It has a high impact on morbidity and a high risk of lethality (43, 44).

The treatment of delirium should be focused on resolving the underlying condition and must be combined with non-pharmacological interventions and specific pharmaceutical interventions. However, the diagnosis of delirium and the early identification of its causal factors depend on the training of the health team. After discharge, patients who have developed delirium will need continuous monitoring (21, 43, 44).

In a public calamity, all healthcare services must be prepared to identify and make the differential diagnosis of delirium, as well as to start treatment immediately. When identifying these cases, health professionals should already have their protocols in place for such assistance (10, 21). Delirium can be caused by different events such as trauma, hydroelectrolytic disorders, complications of pre-existing physical diseases, medications, substance abuse, and infections (especially kidney and lung). For calamities involving infectious diseases, it is important to remember that delirium is part of the set of symptoms and indicates greater severity of the patient's disorder (10).

### Agitation

Psychomotor agitation caused by mental disorders should receive immediate attention. It is important for the clinician to proceed with the mental status examination and consider other psychological processes related to agitation. Despite the existence of psychometric self-report scales to assess agitation, the use of this type of tool is not always viable and can exacerbate agitated behavior (24). In these cases, the use of scales rated by observers could be useful in quantifying the symptoms and assessing the impact of intervention over time (13).

The management of patients, regardless of their environment, must follow the following steps: protection of the patient and people around them, communication and verbal de-escalation, medication approaches, and physical restraint, if all measures fail (13, 24, 28). The Consensus of the American Association of Emergency Psychiatry suggests the use of a de-escalation intervention to address aggressive and agitated patients. This technique includes 10 main domains: (1) respect personal space; (2) do not be provocative; (3) establish verbal contact; (4) be concise; (5) identify wants and feelings; (6) listen closely to what the patient is saying; (7) agree with the patient (agree with the truth, agree with the principle and/or agree with the odds); (8) lay down the law and set clear limits; (9) offer choices and optimism; (10) debrief the patient and staff (28).

The use of medications and physical restraint is recommended in an appropriate environment, such as the emergency room. Therefore, if agitation is so severe that it requires such a strict measure, the patient must be transported by ambulance to the emergency service. The use of medications for agitated patients should follow the principles of rapid tranquilization: medicating without overly sedating and using medications with the fewest possible side effects (30).

In places where patients remain for therapeutic interventions, there should be an observation area with quick access for the health team and support material for evaluation. The medication procedure is called rapid reassurance and requires periodic monitoring of vital signs and the patient's state of consciousness, as well as physical restraint (13, 21).

In calamities, agitated patients often also suffer from physical illnesses; therefore, they need to be managed in health service centers with general medical support, including that for trauma and infectious diseases (10). If there is a risk of contagion, agitated patients, most of whom are uncontrolled in their volition, need to be protected from contact with other people. In such cases, a private approach is essential, since the patients often may not accept the use of personal protective equipment (10).

Cases of psychomotor agitation require quick decisions and can often involve poorly planned and aggressive measures. In turn, in situations of public calamity, patients may not be accurately assessed. Therefore, extra care should be taken with the use of medications. Antipsychotic agents (aripiprazole, olanzapine, quetiapine, risperidone, and haloperidol, among others) are associated with a 1.7- to 3-fold risk of hospitalization due to pneumonia (45) and an increased risk of sudden death and thrombosis. The risk associated with second-generation antipsychotics is not lower than that associated with first-generation agents. These drugs, however, can also cause respiratory dyskinesia that may be mistaken for asthma or other lung conditions and can lead to inappropriate treatment. Benzodiazepines may be related to hypoventilation.

Even in public calamities, the principles of treatment should always be the same as in regular therapy: medicate only if necessary, with the lowest doses necessary to calm the patient (quick reassurance) and always consider side effects and drug interactions. The use of agitation protocols as a reference is recommended (13, 24, 28, 30, 46).

### Suicide Risk

Although care is expected to reduce the rate of new infections or other physical complications, there is always a high risk of suicide during disasters. The secondary consequences of social distancing may increase the risk of suicide. It is important to consider changes in a variety of economic, psychosocial, and health-associated risk factors (19, 21, 38, 39).

Many studies have documented elevated suicide rates, even among medical professionals (47–50). This at-risk group is now serving at the front lines of the battle against COVID-19. A national discussion is emerging about health care workers' concerns about infection, exposure of family members, sick colleagues, shortages of necessary personal protective equipment, overwhelmed facilities, and work stress. This special population deserves support and prevention services.

The therapeutic approach to suicidal behavior, both ideation or attempts, must include the assessment of risk and protective factors associated with intervention measures (called the safety plan) (38, 39, 51, 52). For cases involving a high risk of suicide, strict observation of the individual is required, so they must stay in the emergency department or undergo hospitalization or home care. This last resource should only be used if a support network is present in the community, such as the following: having a family member or other person who constantly watches the patient, quick access to mental health care for complications and monitoring, and acceptance of the caregiver by the patient (10).

The assessment of the patient for suicide risk can include brief psychometric tools but they should never serve as the only source of information since their predictive value is only moderate (53). Clinicians should look beyond psychometry to perform a comprehensive assessment. According to Weber et al. (54), the patient assessment should include the identification of the present level of risk and the modifiable and fixed risk factors related to the intention. The risk (e.g., previous suicide attempt, psychiatric illness, substance dependence) and protective factors (e.g., interpersonal support, positive coping skills) should also be identified (38, 39).

In emergency service centers, patients at risk of suicide must first receive any general health care. Many suicide attempts are associated with severe trauma or intoxication, and such emergencies should not be neglected to assess psychiatric emergencies (they must be carried out together) (38, 39).

Despite these challenges, there are opportunities to improve suicide prevention efforts in this unique time. Maintenance of some existing efforts is also possible and includes the following movements: Physical Distance, Not Social Distance, Tele–Mental Health, Increase Access to Mental Health Care, and Distance-Based Suicide Prevention and Media Reporting (55).

Actions should be taken to mitigate potential unintended consequences on suicide prevention efforts, which also represent a national public health priority.

### Disorders Due to Substance Use

The most common emergencies related to substance use and abuse are acute intoxication, withdrawal syndrome, severe dependence, and induced conditions, such as psychosis. Here, we will present considerations for each situation. An important warning is that many cases of substance abuse occur through medications, mainly psychotropic and narcotic drugs. For this reason, in times of disaster, health professionals and managers should provide tools for controlled and supervised prescription. In addition, many cases of substance abuse can be associated with suicidal behavior, which further reinforces the need for supervision (10, 19, 21).

#### Acute Intoxication

In the assessment of intoxication, basic life support must first be offered, followed by a brief assessment to identify the substance(s) used, followed by the use or not of measures to reduce absorption, increase excretion, or incorporate antidotes. For this, specific protocols must be used.

#### Withdrawal Syndrome

Patients in disaster situations may be more exposed to this syndrome due to abstinence from substances and medications. The protocol of care must include basic life support and specific services for abstinence. After improving patient abstinence, a specific therapeutic approach for addiction should be performed. Patients who are in isolation or hospitalized, due to physical or mental illness, and who are cigarette users need to receive support for nicotine abstinence. Therefore, health services must be prepared to provide nicotine replacement methods, such as patches.

#### Severe Dependence

Emergencies involve the use of substances that put the patient's life at risk. Examples of these situations include severe physical impairment, such as malnutrition, kidney and liver failure, psychotic symptoms, and other induced conditions and suicidal behavior. In serious cases, hospitalization is required. The healthcare system must be prepared to provide support to these patients.

#### Induced Disorders

Substance-induced mental disorders are a priori emergencies, as they represent a severe complication of substance abuse. The treatment, which should include measures aimed at discontinuing the substance, must be focused on the specific treatment of symptoms.

### Psychosis

During calamities, patients that may be the most neglected are those with psychotic disorders. Such diseases interfere with the patient's critical thinking abilities and in turn with adherence to disaster measures. In addition, these patients are very susceptible to stigma and neglect (14). Treatment should be prioritized for the guidance and use of antipsychotics. Special attention must be paid to emergency cases that involve the following: refractoriness of psychotic symptoms associated with agitation or aggression, suicidal behavior, severe physical damage, risk presented to others (10).

Particular attention should be given to patients with schizophrenia since this mental illness is related to the high prevalence of comorbid disorders such as diabetes type II, pulmonary chronic disease, and hypertension/coronary heart disease (56).

Another important concern is outpatient support, which must be available to monitor patient compliance and prevent further outbreaks. Therefore, in addition to an integrated health network, it may be necessary to use medications that hinder adherence, such as long-acting antipsychotics. In cases where there is a need for observation or hospitalization, patients must remain under the care of specialized, protected, and trained staff members (10).

### Mood Disorders

Mood disorders include depressive episodes in depressive and bipolar disorder and manic and mixed episodes in bipolar disorder. The same principles of treatment must be adopted as in psychotic disorders. Drug treatment and emergencies should also be prioritized. In the case of patients being hospitalized for physical illnesses, the use of antidepressant and mood-stabilizing medications, as well as antipsychotics, should be planned while considering their side effects and drug interactions (21, 57).

### Anxiety Disorders

Anxiety disorders may not seem like emergencies, but they are associated with great suffering for patients, and may lead to substance abuse, suicidal behavior, and aggravation of other disorders. Anxiety disorders encompass a wide variety of disorders, including those related to trauma caused by calamities, such as acute reaction to stress and posttraumatic stress disorder. Screening of anxiety disorders for emergency identification must be available to the population. Psychotherapeutic approaches should be prioritized. Long-term drug treatment with antidepressants is more appropriate, but more immediate responses can be obtained with benzodiazepines. However, these medications are related to a higher risk of abuse, dependence, and suicide; therefore, they require strict supervision, as mentioned above (10, 21).

### Special Populations

Some patients have cognitive and intellectual disabilities, while others require special considerations during disasters. Children cannot be kept in the hospital, isolated, or quarantined without caregivers for an extended period. Teenagers may have difficulty adhering to quarantine and isolation rules. Similar to health professionals, adolescents are more likely to break their quarantine (58). Children and adolescents need structured activities and routines. Their routine can be designed to look like the isolation period, or it can be a new routine (58).

Pregnant women also need special attention in cases of isolation or quarantine. Pregnant women may be particularly concerned about the well-being of their babies and the effect that the infection may have on the fetus. Pregnancy itself can cause emotional problems, and the presence of an infectious condition can further complicate the patient's fears (58).

The postpartum period exposes mothers to a greater risk of postpartum depression or postpartum exacerbation of existing mood disorders. Screening, support, and guidance should be provided, since they can have a positive effect on mothers who give birth in isolation (58).

## Future Directions

In light of several tragedies that have occurred throughout history and in recent years, including the current COVID-19 pandemic, it is necessary to point out that during public calamities, an increase in the number of emergencies is to be expected, either due to the stress of the population or the number of health complications and difficulties in accessing care (2, 3, 18). Therefore, the public sector must be prepared to address such situations using the prevention and intervention measures mentioned above. Within this context, the application of guidelines through evidence-based medicine is essential [i.e., (13, 30, 38, 39, 57, 59–61)].

It is important to note that effective public policy should include both the prevention and treatment of mental disorder outbreaks during pandemics. Providing resources and training that involve all levels of health care is necessary. At the primary level, the prevention of mental health disorders includes attention to special populations that have been exposed to stressors during pandemics due, for example, to specific health conditions (8) or if they have been working in situations with a high risk of exposure to COVID-19 (3). However, secondary, and tertiary health care personnel should also be ready to receive patients in emergency situations and with chronic psychiatric conditions. This presents a double challenge of including resources for both professional training and reformulation of the ambulatory and hospital environment to avoid the risk of contagion. Furthermore, a new demand emerges in this situation since a patient in an acute stage of psychiatric illness may resist adopting behaviors needed to prevent contagion, resulting in an additional clinical target during psychiatric emergency care. In this context, the adoption of strategies described in this article should be integrated with specific behavioral strategies used during pandemics.

## Conclusion

In public calamities, patients can present with psychiatric emergencies that require care and cannot be neglected. It is necessary to maintain protocols, trained teams, and appropriate locations for such support. Some emergencies may occur with the aggravation of pre-existing cases, while others may be new cases that arise because of the traumatic event. Therefore, all health services must consider this type of emergency during disasters, whether they are accidents or epidemics. Many countries do not have basic conditions of care for mental health disorders, and may not have places to support such care, both in terms of physical structure and human resources. However, addressing these issues and taking action to provide good mental health care services will facilitate emergency care. Governments and ministries of health should prepare for calamities, and protocols, plans, or programs should be prepared in advance. Additionally, it is advisable to acknowledge the lessons that we have learned from the COVID-19 pandemic so far.

## Author Contributions

All authors listed have made a substantial, direct and intellectual contribution to the work, and approved it for publication.

## Conflict of Interest

The authors declare that the research was conducted in the absence of any commercial or financial relationships that could be construed as a potential conflict of interest.
